# Myasthenia gravis with double-seropositive acetylcholine receptor and low-density lipoprotein receptor-related protein 4 antibodies combined with muscle atrophy: a case report and literature review

**DOI:** 10.3389/fimmu.2025.1545579

**Published:** 2025-04-28

**Authors:** Yue-liang Zheng, Gong-zhang Su, Yan-lin Li, Tong Du, Xue-lu Zhao, Cong-cong Wang, Ying Liu, Bin Liu, Rui-sheng Duan, Xiao-li Li

**Affiliations:** ^1^ Department of Neurology, The First Affiliated Hospital of Shandong First Medical University and Shandong Provincial Qianfoshan Hospital, Jinan, China; ^2^ Department of Thoracic Surgery, The First Affiliated Hospital of Shandong First Medical University and Shandong Provincial Qianfoshan Hospital, Jinan, China; ^3^ Shandong Institute of Neuroimmunology, Jinan, China; ^4^ Shandong Provincial Key Medicine and Health Laboratory of Neuroimmunology, Jinan, China; ^5^ Department of Neurology, Shandong Provincial Third Hospital, Jinan, China

**Keywords:** myasthenia gravis, muscle atrophy, AChR antibody, LRP4 antibody, neurology

## Abstract

**Objective:**

To investigate the clinical characteristics and mechanisms of muscle atrophy in myasthenia gravis (MG) patients who are double-seropositive with acetylcholine receptor (AChR) antibodies and low-density lipoprotein receptor-related protein 4 (LRP4) antibodies.

**Methods:**

The clinical data, imaging characteristics, treatment methods, and prognosis of one case of MG with AChR/LRP4 antibodies complicated by muscle atrophy were analyzed. Literature on anti-AChR/LRP4 antibodies double-seropositive MG with muscle atrophy were reviewed.

**Conclusion:**

Clinically, anti-AChR/LRP4 antibodies double-seropositive MG is rare, often onset after middle age, more common in females, frequently involving bulbar muscles, severe symptoms, poor prognosis, and unrelated to thymoma. Muscle atrophy in MG is not only seen in muscle-specific tyrosine kinase (MuSK)-MG but also in AChR-MG and seronegative MG. The mechanism of muscle atrophy may be related to genetic, immune, and nutritional factors.

## 1 Introduction

Myasthenia gravis (MG) is an autoimmune disease characterized by antibody-mediated, cell-dependent immune response and complement involvement, affecting the postsynaptic membrane of the neuromuscular junction and causing neuromuscular transmission disorders. Pathogenic antibodies include anti-acetylcholine receptor (AChR) antibodies, anti-muscle-specific receptor tyrosine kinase (MuSK) antibodies, and anti-low-density lipoprotein receptor-related protein 4 (LRP4) antibodies. Cases of MG with overlapping positive antibodies are rarely reported, and the specific mechanisms remain unclear. Clinically, MG symptoms mainly manifest as skeletal muscle involvement, characterized by fluctuation and easy fatigability, but concomitant muscle atrophy is relatively rare (1). Literature reports indicate that only 5% to 10% of MG patients may exhibit localized muscle atrophy, which can affect the tongue muscles, facial muscles, neck muscles, and proximal limb muscles. Here, we report a case with double-positive AChR/LRP4 antibodies in MG combined with muscle atrophy, and review the relevant literature.

## 2 Methods

### 2.1 Clinical data

The patient was subjected to detailed clinical examinations, neurophysiological studies, and neuroimaging examinations. Ethical approval was granted by the ethics committee of the First Affiliated Hospital of Shandong First Medical University. Written informed consent was obtained from the patient before the investigation.

### 2.2 The degree and extent of muscle damage and the neuromuscular junction were evaluated by electromyography

Neuroelectrophysiology assessments primarily included the use of a Nihon Kohden MEB-9400 EMG machine from Japan to evaluate neuromuscular junction function and the extent and nature of muscle damage. Surface electrodes were used for nerve conduction studies, which mainly included repetitive nerve stimulation (RNS). Concentric needles were used to perform needle EMG to observe abnormal spontaneous potentials (positive sharp waves and fibrillation potentials). The discharge patterns (activation, recruitment, and interference patterns) of motor unit action potentials (MUAPs) were assessed.

### 2.3 Neuroimaging

Magnetic resonance imaging (MRI) evaluations were performed on the thymus and proximal muscles of the lower limbs. MRI was conducted at a field strength of 3.0T using a Siemens MAGNETOM Skyra. Scanning included various sequences (T1-weighted imaging, T2-weighted imaging, T2-FLAIR, enhanced T1, enhanced T2-FLAIR, T2 STIR, in-phase, and out-of-phase).

### 2.4 Literature review

The term “muscle atrophy”, “myasthenia gravis”, “AChR antibody” and “LRP4 antibody” were searched in pubmed database, and the similarities and differences in clinical data in “double-seropositive AChR/LRP4 antibodies MG with muscle atrophy” were analyzed (**Table 1**).

**Table 1:** Characteristics of the present and previously reported AChR-MG patients with LRP4 antibodies.

	No. of cases	Ref	Age	gender	MG related antibodies			Symptoms	Thymus	MGFA	EMG	amyotrophy
					AChR	MuSK	LRP4					
P1	1 case	Hidehiro Ishikawa, et al (4)	74	F	+	–	+	ptosis, dysarthria, dysphagia, respiratory failure	Thymoma (B2-3)	IIIb	RNS (+)	NF
P2	1 case	Hidehiro Ishikawa, et al (4)	64	F	+	–	+	ptosis, dysarthria, dysphagia, respiratory failure	Thymoma (B3)	IIIb	RNS (+)	NF
P3	1 case	Tsivgoulis G, et al (3)	67	M	+	–	+	Dysarthria, Dysphagia	Normal	ND	RNS (-)	NF
P4	1 case	Cossins J, et al (31)	ND	ND	+	–	+	ND	ND	ND	ND	ND
P5	8 cases	Zisimopoulou P, et al (2)	ND	ND	+	–	+	ND	Hyperplasia:4/6 Involuted: 2/6	II:1/8 III:7/8	ND	ND
P6	3 cases	Marino M, et al (32)	ND	ND	+	–	+	ND	ND	ND	ND	ND
P7	7 cases	Nikolic AV, et al (7)	34-51	M : F = 3:4	+	–	+	ND	ND	IIIB: 4/7 IVB: 2/7 V: 1/7	RNS (+): 5/7	ND
P8	1 case	Suresh C. Bokoliya, et al (6)	32	M	+	+	+	Limbs weakness	Normal	IIIa	amyotrophy	Nasalis, abductor digitiminimi muscles
P9	1 case	Hiroyasu Inoue, et al (5)	37	M	+	–	+	ptosis, diplopia dysarthria, dysphagia → hypercapnic respiratory failure → Intubation	Thymic Hyperplasia	V	RNS (+)	NF
P10	1 case	Present study	74	M	+	–	+	ptosis, dysphagia → hypercapnic respiratory failure → Intubation	Thymic Hyperplasia	V	RNS (-), Muscular damage	Dorsal muscles between the first and second bones of Hyperplasia respiratory failure →Intubation both hands, thenar, scapular, thigh muscle

F, female; M, male; AChR, acetylcholine receptor; MuSK, muscle-speciffc receptor tyrosine kinase; LRP4, LDL Receptor-Related Protein 4; Thymoma (B2-3), Thymoma of WHO type B (B2 with areas of B3); Thymoma (B3), Thymoma of WHO type B3; MGFA, Myasthenia Gravis Foundation of America; RNS, repetitive nerve stimulation; EMG, electromyography; ND, Not done; NF, not found.

## 3 Results

### 3.1 Case presentation

A 74-year-old male patient (with a 35-year history of diabetes mellitus) presented in 2019 with left eyelid ptosis, which worsened in the evening and improved following periods of rest. The condition developed without any identifiable cause, and the patient did not seek medical evaluation at that time. In June 2020, the patient sought medical attention at an external hospital for diabetes management. During the physical examination, no muscle atrophy was noted. Body composition analysis revealed reduced skeletal muscle mass in both upper limbs and the trunk. Nerve conduction studies of the lower extremities demonstrated slowed sensory nerve conduction velocities in the bilateral tibial nerves, primarily indicative of axonal damage, consistent with diabetic peripheral neuropathy. The patient’s muscle strength in all four limbs was normal, and no EMG was performed at that time. Mild atrophy of the first dorsal interosseous muscles of both hands was observed.

In March 2021, following a cold, he developed respiratory distress and dysphagia, along with limb weakness, difficulty in lifting his head, coughing, sputum production, and dyspnea. The patient was admitted to the respiratory department. His condition gradually deteriorated. Due to respiratory failure, tracheal intubation and mechanical ventilation were initiated on March 21, 2021. A consultation was then requested from the neurology department for an assessment. Serum antibody testing revealed positive results for AChR antibodies (1:320) and LRP4 antibodies (1:10), with negative results for MuSK antibodies, Titin antibodies, and ryanodine receptor (RyR) antibodies (Beijing-Tianjin Neuroimmunology Center Innovation and Translational Laboratory, cell-based assay). Due to the rapid progression of the patient’s condition, the neostigmine test and EMG were not performed. Based on the clinical presentation, serological findings, and the characteristic improvement in symptoms following neostigmine administration, a diagnosis of MG was made. The patient received intravenous immunoglobulin (IVIg), steroid pulse followed by a gradual taper (500 mg daily for 3 days, gradually decrease to 60 mg), and anti-infection therapy based on the sputum culture results (cefoperazone-sulbactam sodium, imipenem in combination with vancomycin, and other appropriate antibiotics). To manage MG further, tacrolimus at an initial dose of 1 mg three times daily was administered for 10 days, achieving an concentration of 1.8 ng/ml. Tacrolimus dosage was subsequently adjusted to 2 mg twice daily. After 11 days of mechanical ventilation in early April 2021, the patient was successfully extubated while maintaining limb movement. At discharge, the patient had a normal body temperature, occasional coughing without expectoration, no ptosis, dysphagia, or aspiration while eating, and could ambulate independently without limitations in daily activities. In mid-April 2021, the patient gradually developed dysphagia, limb weakness, difficulty turning over and getting out of bed, squatting and standing, and required assistance to walk. Physical examination revealed coarse breath sounds in both lungs with few moist rales, muscle atrophy in the first and second dorsal interosseous muscles of both hands, thenar muscles, bilateral scapular muscles, and proximal muscles of both lower limbs. He exhibited bilateral weak cheek puffing, dysphagia, weak head lift but no tongue muscle atrophy. The Quantitative Myasthenia Gravis (QMG) score was 14. EMG conducted two hours after pyridostigmine bromide administration showed no abnormalities in RNS, but direct muscle stimulation indicated myogenic damage. Flow cytometry analysis revealed: mature T cells 86.21% of lymphocytes, Th cells 53.42% of lymphocytes, CTL cells 30.29% of lymphocytes, Treg cells 4.46% of lymphocytes, NK cells 5.13% of lymphocytes, and B lymphocytes 8.22% of lymphocytes. Serological tests showed IgG (9.46 g/L), IgA (1.03 g/L), and IgM (0.715 g/L) were all within the normal range. The immune function of the patient was considered to be reasonable. Serum lactate dehydrogenase level was 355U/L, and creatine kinase level was within the normal range. Serum myositis antibody panel negative for anti-Jo-1 antibody, anti-PL-7 antibody, anti-PL-12 antibody, EJ, OJ, anti-MI-2α antibody, anti-MI-2β antibody, anti-MDA5 antibody, anti-TIF1γ antibody, anti-NXP2 antibody, anti-SAE1 antibody, SRP, anti-RO-52 antibody, anti-PM-SCL75 antibody, anti-PM-SCL100 antibody, and anti-KU antibody, and without myalgia or elevated creatine kinase levels, ruling out myositis. On April 30, 2021, EMG, re-evaluated 20 hours after discontinuation of pyridostigmine bromide demonstrated no significant changes in MUAPs compared to the previous examination, and RNS remained negative. Treatment with prednisone acetate, tacrolimus (2.5 ng/ml), and pyridostigmine bromide led to gradual clinical improvement and subsequent discharge.

**Figure 1:**
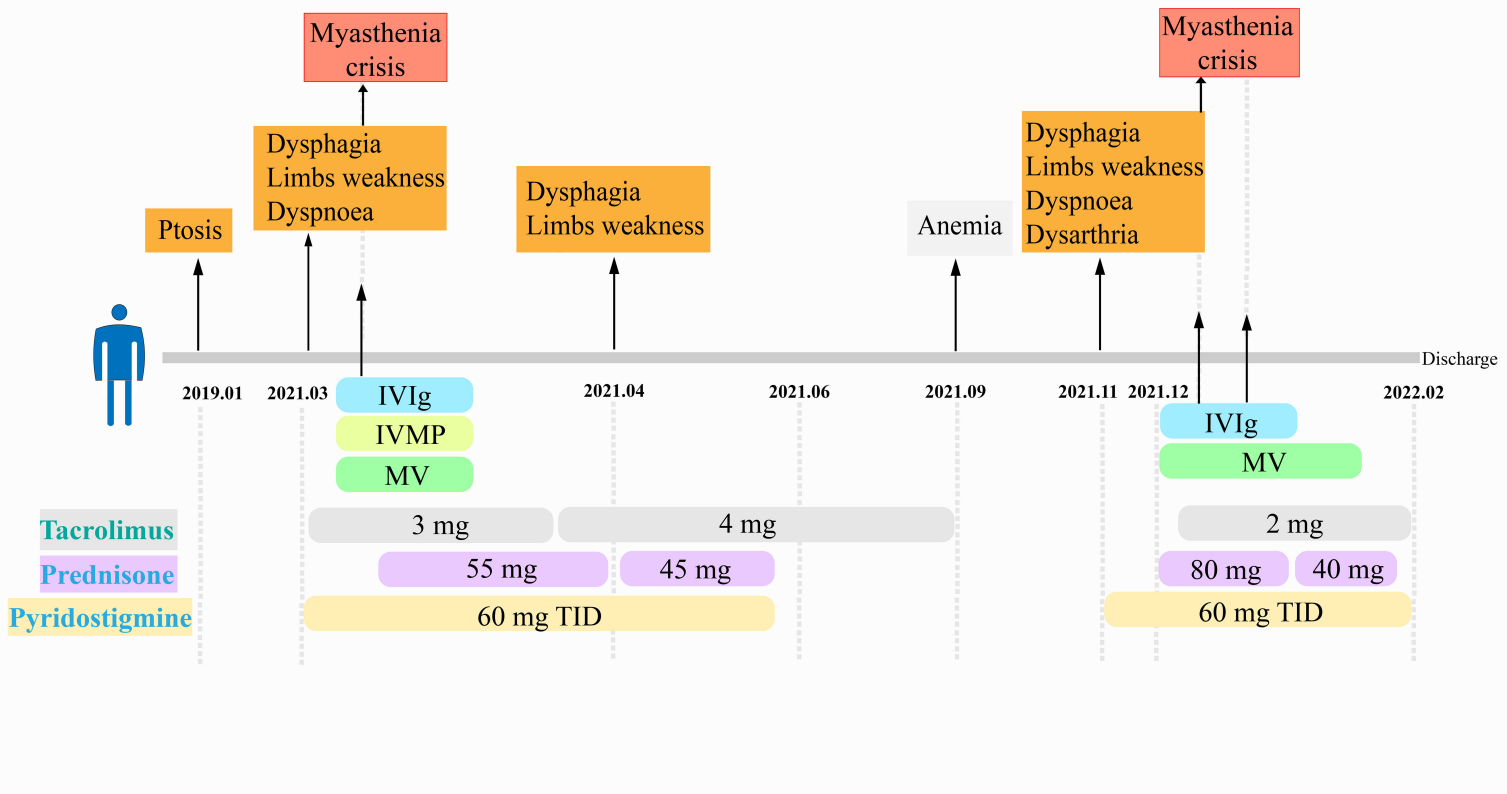
The clinical course after the onset. IVIg, intravenous immunoglobulin; IVMP, intravenous methylprednisolone; MV, mechanical ventilation; TID, three injections daily.

On December 7, 2021, the patient developed bilateral eyelid ptosis, diplopia, dysarthria, weak cheek puffing, dysphagia, choking while drinking, and weak head lift following an upper respiratory tract infection. The condition rapidly progressed to a myasthenic crisis with a QMG score of 22, necessitating tracheal intubation and mechanical ventilation. On December 8th and December 20th, 2021, two rounds of IVIg therapy were administered. Methylprednisolone was initiated at 80 mg/day and gradually tapered to 40 mg/day. Tacrolimus was administered at 1 mg twice daily. Based on sputum culture results, piperacillin-tazobactam sodium and cefoperazone-sulbactam sodium were sequentially used for antimicrobial treatment. The patient was successfully weaned off ventilation and discharged in a stable condition (tracheostomy, generalized emaciation, upper limb muscle strength grade 4, lower limb muscle strength grade 3). Post-discharge medication included pyridostigmine bromide 60 mg three times daily, prednisone acetate 30 mg once daily, and Tacrolimus 1 mg twice daily. Despite these interventions, his prognosis was poor, and he died of respiratory failure outside the hospital. (**Figure 1**).

### 3.2 EMG

An EMG examination was conducted two hours after pyridostigmine administration on April 16, 2021. Low-frequency RNS tests showed no decrement exceeding 15% in the amplitude of the compound muscle action potential, while high-frequency RNS tests showed no increment in the amplitude. Specifically, the amplitude of the 3 Hz stimulation of the left ulnar nerve decreased by 4.4% and for the right accessory nerve, it decreased by 2.1% at 3 Hz but increased by 0.2% at 50 Hz). Needle EMG revealed acute denervation potentials in the right tibialis anterior, bilateral first dorsal interossei, and left extensor digitorum communis muscles. Short-duration MUAPs with increased recruitment were observed during minimal voluntary contractions in the bilateral tibialis anterior, right medial gastrocnemius, bilateral biceps brachii, right iliacus, left extensor digitorum communis, and left extensor indicis muscles. Increased polyphasic waves were seen in minimal voluntary contractions of the aforementioned muscles except for the right first dorsal interosseous muscle. Direct muscle stimulation responses indicated myogenic damage with an amplitude ratio close to 1.

Considering the potential influence of pyridostigmine bromide and the patient’s ability to tolerate the examination, an EMG was repeated 20 hours after discontinuing pyridostigmine bromide on April 30, 2021. The EMG revealed increased polyphasic waves during low-force contractions in the right biceps brachii, medial head of the right quadriceps femoris, right iliacus, right tibialis anterior, and left abductor digiti minimi muscles. Pathological interference patterns were observed in the right biceps brachii, medial head of the right quadriceps femoris, and right iliacus, suggesting myogenic damage. RNS of the right ulnar nerve showed a decremental response of 0.5% at 3 Hz, 2.9% at 10 Hz, and 12.2% at 20 Hz, with incremental responses of 2.1% at 30 Hz and 3.7% at 50 Hz. The left accessory nerve exhibited a 4.3% increment at 3 Hz, while the left facial nerve demonstrated a 1.7% decrement at 3 Hz. Motor and sensory conduction velocities of the bilateral median and ulnar nerves were reduced, predominantly indicating axonal damage, consistent with peripheral neuropathy. MUAPs remained unchanged in comparison with the results prior to pyridostigmine bromide administration, and RNS results were negative.

### 3.3 Neuroimaging

**Figure 2:**
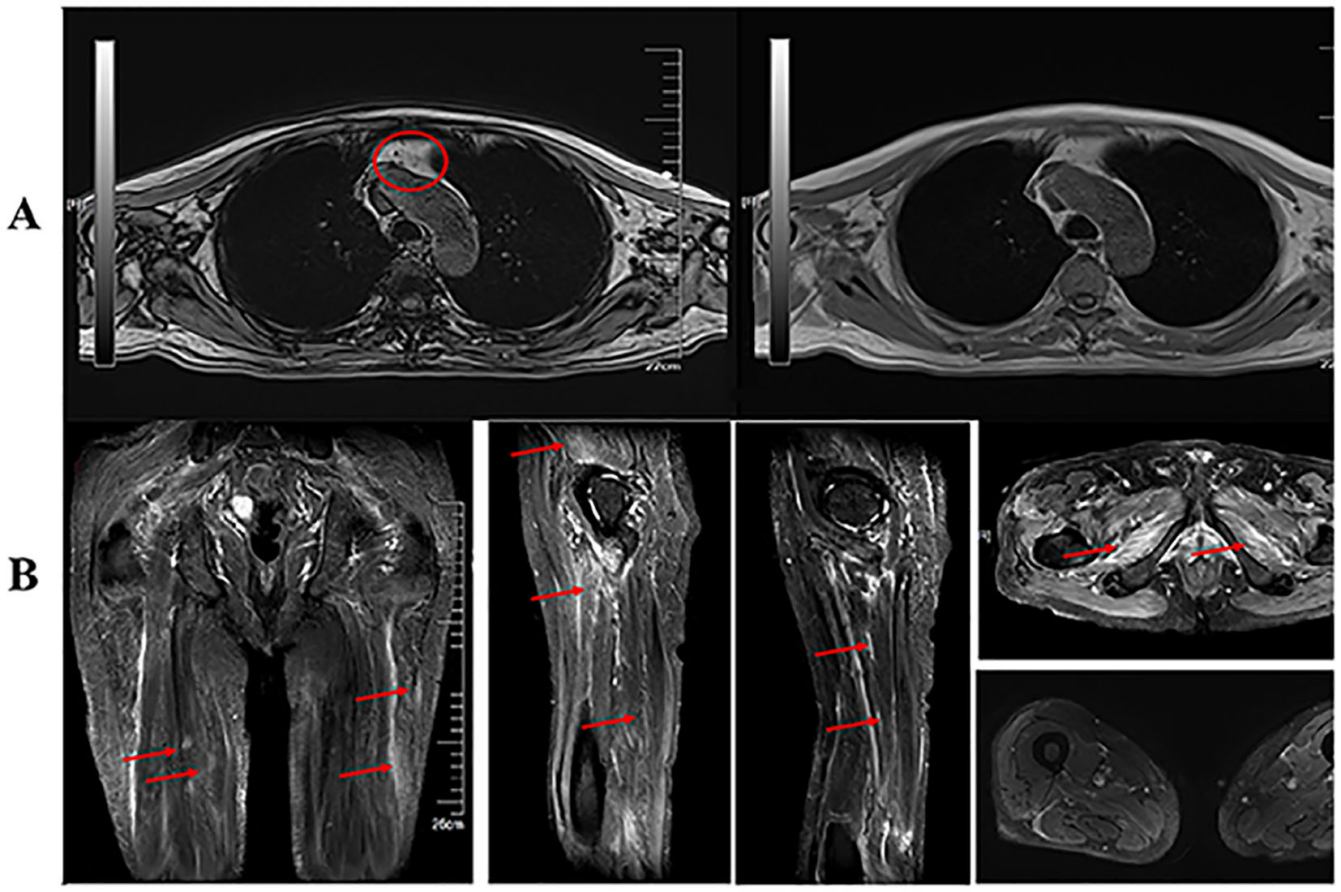
**(A)** Magnetic resonance imaging (MRI) of the patient’s thymus revealed a slight narrowing of the antiphase area in the mediastinum thymus region, and no abnormalities were seen on enhanced CT. **(B)** There were no abnormal lymph nodes in the hilum or mediastinum. This was considered to be thymic hyperplasia. Peripelvic floor muscles, bilateral buttocks and bilateral thighs showed diffuse hyperintense T2 fat-suppression signals. Some of the muscles were mildly atrophied.

Thymic MRI indicated mild hypointensity in the thymic region of the anterior mediastinum on out-of-phase imaging, suggesting possible thymic hyperplasia. Bilateral thigh MRI showed diffuse patchy T2-weighted fat-suppressed hyperintensities in the muscles of the pelvic floor, bilateral gluteal, and bilateral thigh regions, with mild atrophy in some muscles (**Figure 2**).

### 3.4 Diagnosis

Based on clinical symptoms and auxiliary examinations, the patient was diagnosed with AChR/LRP4 antibodies double-seropositive MG, complicated by thymic hyperplasia, and concomitant muscle atrophy in the first and second dorsal interossei, thenar muscles, bilateral scapular muscles, and proximal muscle groups of the lower limbs.

## 4 Discussion

The prevalence of MG patients who are double-seropositive for AChR/LRP4 antibodies is exceptionally low. Zisimopoulou et al. reported eight cases of MG patients who were positive for both AChR and LRP4 antibodies (2), Among these, five patients exhibited bulbar muscle involvement, seven were classified as MGFA type III, one experienced a myasthenic crisis, and three developed muscle atrophy. All patients achieved favourable outcomes following immunotherapy. Tsivgoulis et al. described a male patient with double-positive AChR/LRP4 antibodies who presented solely with bulbar muscle symptoms and responded effectively to prednisone treatment (3). Ishikawa. reported two cases of AChR/LRP4-positive MG associated with invasive thymoma. Although the initial symptoms were limited to ocular muscles, the disease eventually progressed to MGFA class III. Both patients had acceptable prognoses after appropriate treatment. In summary, patients with MG who are double-positive AChR/LRP4 antibodies exhibit more severe conditions, often involving the bulbar and systemic muscle groups (4).

Hiroyasu Inoue et al. identified a patient with autoimmune polyglandular syndrome (APS) who was positive for both AChR/LRP4 antibodies, as well as APS antibodies. This patient displayed more severe clinical symptoms, suggesting that APS-related autoantibodies, in conjunction with LRP4 antibodies, exacerbated the patient’s condition (5). Suresh C. Bokoliya et al. reported a case positive for AChR, LRP4, and MuSK antibodies, suggesting that the coexistence of two or more antibodies might be related to immune dysregulation or cross-reactivity, consistent with the epitope spreading mechanism. This mechanism involves autoimmune attacks on synaptic epitopes leading to post-synaptic AChR damage. These epitopes are then processed by antigen-presenting cells, generating new immunogenic determinants, which ultimately result in the production and expression of multiple antibodies (6). To date, a total of 25 cases of MG with double positivity for AChR/LRP4 antibodies were reported, including our case. Among the 20 cases with described MGFA classification: 3 cases (15%) were type V, 2 cases (10%) were type IV, 14 cases (70%) were type III, and 1 case (5%) was type II, all of which were generalized MG. Of the 12 cases with reported gender, the male-to-female ratio was 1:1, with most of these cases being of early-onset or very late-onset. Initial symptoms often involved ocular and bulbar muscles, with rare initial involvement of limb muscles. Most patients eventually developed respiratory difficulties accompanied by worsening ocular and bulbar muscle symptoms, and some even experienced myasthenic crises. Among the 10 reported cases with both AChR/LRP4 antibodies and thymic abnormalities, 2 cases (20%) had thymoma, and 6 cases (60%) had thymic hyperplasia. Among 11 cases underwent RNS examination, 8 cases (72.7%) showed positive RNS results. According to the literature, the positive rate of RNS in patients with MG who are double-positive for AChR/LRP4 antibodies is significantly higher than that observed in patients who are double-positive for MuSK/LRP4 antibodies. However, the RNS positive rate in AChR/LRP4 double-positive MG patients is lower compared to patients who are single-positive for anti-AChR antibodies (7). RNS of our case was normal. Case 4 and our case exhibited muscle atrophy. muscle atrophy in our case involved the first and second dorsal interossei of both hands, thenar muscles, bilateral scapular muscles, and proximal muscle groups of the lower limbs. Case 4 had atrophy involving the nasalis and abductor digiti minimi muscles. Other reports did not indicate the presence of muscle atrophy.

Myasthenia gravis (MG) associated with muscle wasting is typically seen in the following conditions: (1) MG associated with myositis; (2) steroid myopathy; (3) critical illness myopathy (CIM); (4) MG in conjunction with muscular atrophy. Due to limitations in performing muscle biopsy, muscle pathology was not available. The patient has no history of myalgia and normal creatine kinase levels. In addition, all myositis-related antibodies were negative, which does not support the diagnosis of myositis. Steroid myopathy is a muscle disorder resulting from prolonged or high-dose administration of glucocorticoids. It is primarily characterized by proximal muscle weakness and atrophy, the severity of which is positively correlated with both the dosage and duration of glucocorticoid therapy. The patient exhibited muscle atrophy prior to the initiation of glucocorticoid therapy. Therefore, the diagnosis of steroid myopathy can be ruled out. This patient has a documented history of intensive care unit (ICU) admission, thus necessitating the exclusion of CIM. CIM is a prevalent acquired neuromuscular disorder in ICU patients, often coexisting with critical illness polyneuropathy (CIP). It is characterized by the rapid onset of symmetrical weakness affecting both proximal and distal limb muscles, accompanied by muscle atrophy developing over days to weeks. The recovery period from CIM is generally protracted, often spanning weeks to months. In this case, the patient exhibited muscle atrophy prior to ICU admission and maintained limb movement during their ICU stay, which does not align with the diagnostic criteria for CIM. Diabetes mellitus is a significant factor contributing to skeletal muscle atrophy. The mechanisms underlying diabetes-induced skeletal muscle damage are as follows: (1) Inhibition of the IRS-PI3K-AKT-mTOR signaling cascade: Insulin resistance leads to weakened activation of mTOR, resulting in reduced protein synthesis and increased protein degradation in skeletal muscle (8). (2) Mitochondrial dysfunction and reactive oxygen species (ROS) accumulation: In diabetic patients, decreased levels of mitochondrial fusion proteins mitofusin-1 and mitofusin-2 impair mitochondrial function, leading to excessive production of ROS. This results in oxidative stress that damages mitochondrial DNA and the nuclei of skeletal muscle cells, accelerating apoptosis (9). (3) Dysfunction of muscle satellite cells: Impaired myogenic capacity of muscle satellite cells contributes to skeletal muscle atrophy (10, 11). (4) Intracellular fat deposition: Accumulation of lipids within muscle cells further exacerbates muscle dysfunction (12, 13). (5) Skeletal muscle inflammation: Elevated levels of pro-inflammatory cytokines such as interleukin-6 (IL-6), tumor necrosis factor-alpha (TNF-α), and C-reactive protein in diabetic patients inhibit insulin receptor signaling, reducing insulin sensitivity and promoting muscle atrophy (14). Given this patient’s long-standing history of diabetes, it is essential to differentiate his condition from diabetic myopathy. Diabetic myopathy is common in patients with suboptimal glycemic control. It is primarily characterized by proximal muscle involvement, which manifests as weakness in the affected muscles. EMG features of diabetic myopathy typically include predominantly neurogenic changes, characterized by spontaneous potentials during rest, high-amplitude and wide-duration MUAPs during contraction, and the presence of satellite potentials in chronic stages. Additionally, there is a significant reduction in the amplitude of compound muscle action potentials (CMAPs) in proximal lower limb nerves, primarily due to axonal damage. In contrast, this patient’s EMG shows low-amplitude MUAPs and pathological interference patterns, indicative of myogenic damage. Moreover, the widespread decrease in CMAP amplitudes cannot be adequately explained by diabetic complications alone. Therefore, diabetic myopathy can be excluded as a diagnosis.

Oosterhuis (15) reported that 12 out of 418 MG cases had localized tongue muscle atrophy. De Assis (16) et al. conducted a study on 752 MG cases and found that only 10 cases (1.3%) exhibited muscle atrophy. The majority of these cases involved facial, tongue, masticatory, and neck muscles, potentially affecting the tongue, facial, neck, and proximal limb muscles, with the highest incidence in tongue muscles. Tongue muscle atrophy is characterized by the presence of three longitudinal grooves when the tongue is extended, known as “trident tongue”, and isolated limb involvement is rare. According to Johns (17) et al, muscle atrophy is more common in moderate to severe MG cases or in those with a disease duration exceeding one year. This atrophy is often accompanied by a loss of responsiveness to anticholinesterase in the affected muscles. Literature reports indicate that tongue muscle atrophy related to MG is more frequently seen in MuSK-MG. Muscle atrophy in MuSK-MG is not only prone to affect facial and tongue muscles but also bulbar and neck muscles, and the condition tends to be more severe. Our previous case series found that muscle atrophy in MG is not exclusive to MuSK-MG but also occurs in AChR-MG and seronegative MG, with tongue muscle atrophy being the most common form. Most cases of tongue muscle atrophy are reversible with immunotherapy, leading to clinical improvement, and there is a certain correlation between tongue muscle atrophy and bulbar muscle involvement (18).

In MG, muscle atrophy may be associated with gene products related to atrophy, including atrogin-1 and muscle-specific ring finger protein-1 (MURF-1). Benveniste et al. injected the serum of MuSK-MG into C57B16 mice, the expression of MURF-1 was found to be increased in mouse C2C12 cells and in the facial muscles of mice within a short period of time. Within a short time frame, there was an increase in MURF-1 expression in both mouse C2C12 cells and facial muscles (19). This suggests that the serum of MuSK-MG can influence the expression of proteins related to the development of muscle atrophy, with facial muscles being the most sensitive. Research has demonstrated that the IGF1/Akt signaling pathway can inhibit protein degradation and enhance protein synthesis. Downregulation of this pathway promotes muscle atrophy. The absence or dysfunction of key genes in the IGF1/Akt/mTOR signaling cascade exerts a negative regulatory effect on muscle contractility and mitochondrial homeostasis (20). Studies have shown that muscle biopsies from patients with myasthenia gravis-associated muscle atrophy predominantly exhibit type II fiber atrophy, with minimal involvement of type I fibers (21, 22).

Oosterhuis (15) et al. reported 14 cases of MG with muscle atrophy and found that even in the absence of clinical muscle atrophy, neurogenic changes occur in the muscles of MG cases. Brownell (23) and Sanders (24) both observed that MG with tongue muscle atrophy manifests as severe neurogenic atrophy, with fatty pseudohypertrophy and significant peripheral nerve fiber proliferation. Oosterhuis (15) performed biopsies on the limb muscles of MG cases, revealing neurogenic changes, type II muscle fiber atrophy, and lymphocyte infiltration, suggesting that muscle abnormalities in MG cases are due to acetylcholine deficiency and denervation at the neuromuscular junction. Martignago (25) et al. grouped patients based on AChR and MuSK antibodies, finding that muscles in AChR-MG are more prone to neurogenic changes, while MuSK-MG exhibit myogenic changes with significant mitochondrial abnormalities. Both AChR-MG and MuSK-MG exhibit varying degrees of mitochondrial stress and damage. Notably, despite having fewer mitochondria, type II fibers are also affected in these conditions (26). Since ocular and bulbar muscles are high-metabolic muscle groups rich in mitochondria, mitochondrial abnormalities in MuSK-MG contribute to the prominent involvement of ocular and bulbar muscles. Giovanna (27) et al. showed that myopathy and mitochondrial abnormalities are more pronounced in MuSK-MG, characterized by mitochondrial swelling, degradation, and cristae disruption. In AChR-MG, the most common muscle changes include fiber atrophy, muscle fiber disarray, and Z-line streaming, consistent with mild neurogenic abnormalities. Besides genetic regulatory mechanisms, the mechanisms causing muscle atrophy may include: (1) AChR antibodies binding to receptors, blocking the transmission of signals at the neuromuscular junction, also known as “synaptic denervation,” leading to neurogenic muscle atrophy; (2) immune responses causing muscle fiber destruction, resulting in myogenic atrophy; (3) disuse atrophy; (4) coexisting autoimmune diseases such as myositis leading to muscle atrophy.

In AChR-MG, muscle fiber atrophy is prevalent regardless of clinically visible muscle atrophy, involving both type I and type II muscle fibers. EMG and muscle MRI can serve as important tools to distinguish between neurogenic and myogenic muscle atrophy. Some case reports (28) have utilized MRI to study the characteristics of muscle atrophy in MG, revealing significant fat infiltration in the atrophied tongue muscles of AChR-MG patients. Sanadze (29) reported 8 cases of AChR-MG with muscle atrophy, with six showing symmetrical atrophy of the forearms while retaining hand muscles, and two showing scapular muscle atrophy. The atrophy appeared 3–18 years after the onset of MG, suggesting the presence of other neuromuscular diseases causing the atrophy, with MG exacerbating the progression due to neuromuscular transmission impairment.

Is muscle atrophy in MG reversible? Ercikan (30) reported a case where muscle atrophy in an MG patient resolved five months after intensive immunotherapy (including IVIg, plasmapheresis, and tacrolimus) initiated seven months post-symptom onset, highlighting the effectiveness of early intensive immunotherapy. Some reports suggest that myogenic changes are more common in facial muscles after long-term steroid use, but some patients in our cohort developed muscle atrophy before or shortly after steroid use, indicating that muscle atrophy is not entirely related to steroid treatment. Following immunosuppressive therapy, muscle atrophy in these patients improved significantly (18).

## 5 Conclusion

Muscle atrophy in MG is rare, and it is even rarer in MG patients who are sera-positive for both AChR/LRP4 antibodies. From the summary of the case characteristics described above, it is evident that the initial symptoms of such patients vary, but their condition rapidly progresses to involve bulbar muscles, with severe impairment of swallowing function. Respiratory muscle function is easily compromised, leading to breathing difficulties and myasthenic crisis. Muscle atrophy significantly worsen their speech, chewing, movement, and overall quality of life. Conventional treatments often fail to provide effective control, making the initiation of more robust immunotherapy crucial for improving patient prognosis.

### 5.1 Limitations of the study

This is a case report and literature review; the results and conclusions presented herein may not be generalizable for patients who were analyzed MG with AChR/LRP4 antibodies complicated by muscle atrophy. Due to the paucity of relevant case reports, The specific manifestations of myasthenia gravis with double-sero-positive AChR/LRP4 antibodies combined with muscle atrophy cannot be clarified. We still need more relevant cases to be further summarized.

## Data Availability

The original contributions presented in the study are included in the article/supplementary material. Further inquiries can be directed to the corresponding author.
